# Supplementary material for: First Investigation of the Optimal Timing of Vaccination of Nile Tilapia (Oreochromis niloticus) Larvae against Streptococcus agalactiae
Source: Vaccines (Basel). 2023 Nov 24;11(12):1753. doi: 10.3390/vaccines11121753 (PMC10747866; doi:10.3390/vaccines11121753)
Supplement: Supplementary file 1 [file vaccines-11-01753-s001.zip › vaccines-2657163-supplementary.pdf]

Supplement material Table S1.

Time after Vaccination (h)	Group	Weight (g)						
		Day 1	Day 7	Day 14	Day 21	Day 28	Day 35	Day 42
**0**	Control	0.011 ± 0.014	0.039 ± 0.016	0.053 ± 0.011	0.110 ± 0.108	0.330 ± 0.123	0.490 ± 0.128	0.580 ± 0.132
	Treatment	0.011 ± 0.014	0.037 ± 0.018	0.052 ± 0.010	0.108 ± 0.110	0.330 ± 0.131	0.480 ± 0.111	0.580 ± 0.128
**6**	Control	0.012 ± 0.012	0.039 ± 0.015	0.053 ± 0.019	0.120 ± 0.132	0.320 ± 0.121	0.480 ± 0.161	0.590 ± 0.132
	Treatment	0.011 ± 0.010	0.037 ± 0.011	0.051 ± 0.021	0.112 ± 0.168	0.330 ± 0.137	0.480 ± 0.223	0.570± 0.121
**24**	Control	0.011 ± 0.018	0.038 ± 0.013	0.054 ± 0.017	0.110 ± 0.142	0.320 ± 0.150	0.480 ± 0.164	0.590 ± 0.110
	Treatment	0.011 ± 0.011	0.038 ± 0.017	0.051 ± 0.019	0.110 ± 0.133	0.330 ± 0.121	0.480 ± 0.158	0.570 ± 0.115
**168**	Control	0.025 ± 0.023	0.049 ± 0.016	0.071 ± 0.014	0.260 ± 0.154	0.490 ± 0.139	0.560 ± 0.190	0.620 ± 0.184
	Treatment	0.028 ± 0.024	0.052 ± 0.011	0.079 ± 0.016	0.310 ± 0.146	0.542 ± 0.142	0.641 ± 0.165	0.731 ± 0.211
**336**	Control	0.036 ± 0.022	0.052 ± 0.011	0.098 ± 0.027	0.320 ± 0.122	0.540 ± 0.220	0.620 ± 0.134	0.750 ± 0.123
	Treatment	0.038 ± 0.027	0.059 ± 0.021	0.103 ± 0.014	0.450 ± 0.231	0.641 ± 0.194	0.730 ± 0.207	0.821 ± 0.190

Supplement material Table S2.

Time after Vaccination (h)	Group	Total length (cm)						
		Day 1	Day 7	Day 14	Day 21	Day 28	Day 35	Day 42
**0**	Control	0.62 ± 0.05	0.97 ± 0.1	1.25 ± 0.1	1.36 ± 0.1	1.48 ± 0.1	1.62 ± 0.1	1.76 ± 0.1
	Treatment	0.61 ± 0.05	0.95 ± 0.1	1.25 ± 0.1	1.37 ± 0.1	1.45 ± 0.1	1.61 ± 0.1	1.74 ± 0.1
**6**	Control	0.64 ± 0.05	0.99 ± 0.1	1.24 ± 0.1	1.36 ± 0.1	1.48 ± 0.1	1.63 ± 0.1	1.74 ± 0.1
	Treatment	0.63 ± 0.05	0.96 ± 0.1	1.25 ± 0.1	1.37 ± 0.1	1.45 ± 0.1	1.63 ± 0.1	1.74 ± 0.1
**24**	Control	0.63 ± 0.05	0.98 ± 0.1	1.25 ± 0.1	1.37 ± 0.1	1.49 ± 0.1	1.63 ± 0.1	1.76 ± 0.1
	Treatment	0.63 ± 0.05	0.95 ± 0.1	1.25 ± 0.1	1.37 ± 0.1	1.45 ± 0.1	1.63 ± 0.1	1.74 ± 0.1
**168**	Control	0.89 ± 0.1	1.21 ± 0.2	1.32 ± 0.2	1.41 ± 0.2	1.62 ± 0.2	1.73 ± 0.2	1.86 ± 0.2
	Treatment	0.92 ± 0.1	1.31 ± 0.1	1.43 ± 0.1	1.56 ± 0.1	1.72 ± 0.1	1.83 ± 0.1	2.01 ± 0.2
**336**	Control	1.10 ± 0.6	1.29 ± 0.2	1.39 ± 0.1	1.49 ± 0.2	1.73 ± 0.1	1.83 ± 0.2	1.94 ± 0.2
	Treatment	1.30 ± 0.1	1.42 ± 0.1	1.59 ± 0.1	1.67 ± 0.1	1.84 ± 0.1	1.97 ± 0.2	2.18 ± 0.2

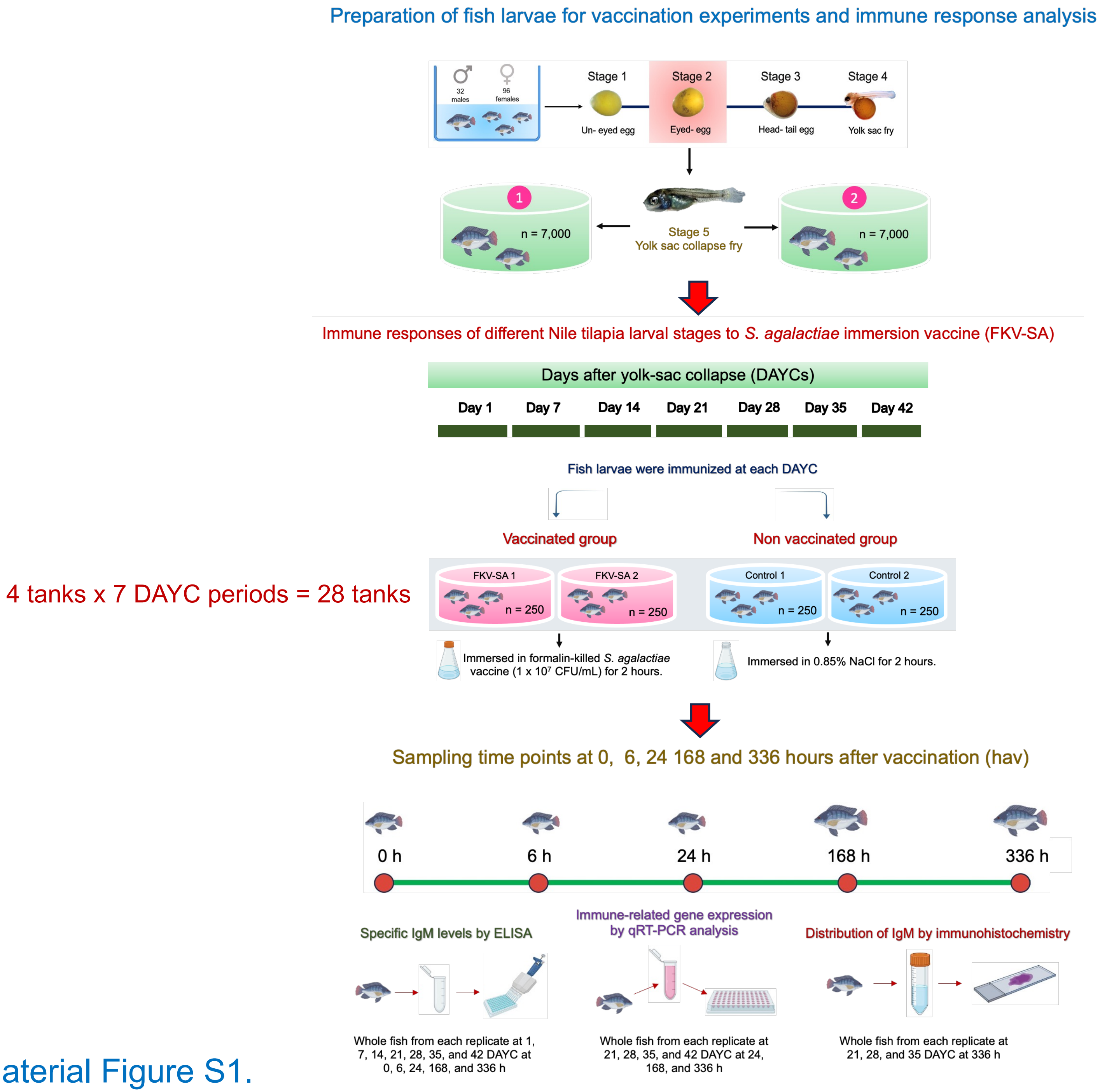

Supplement material Figure S1.

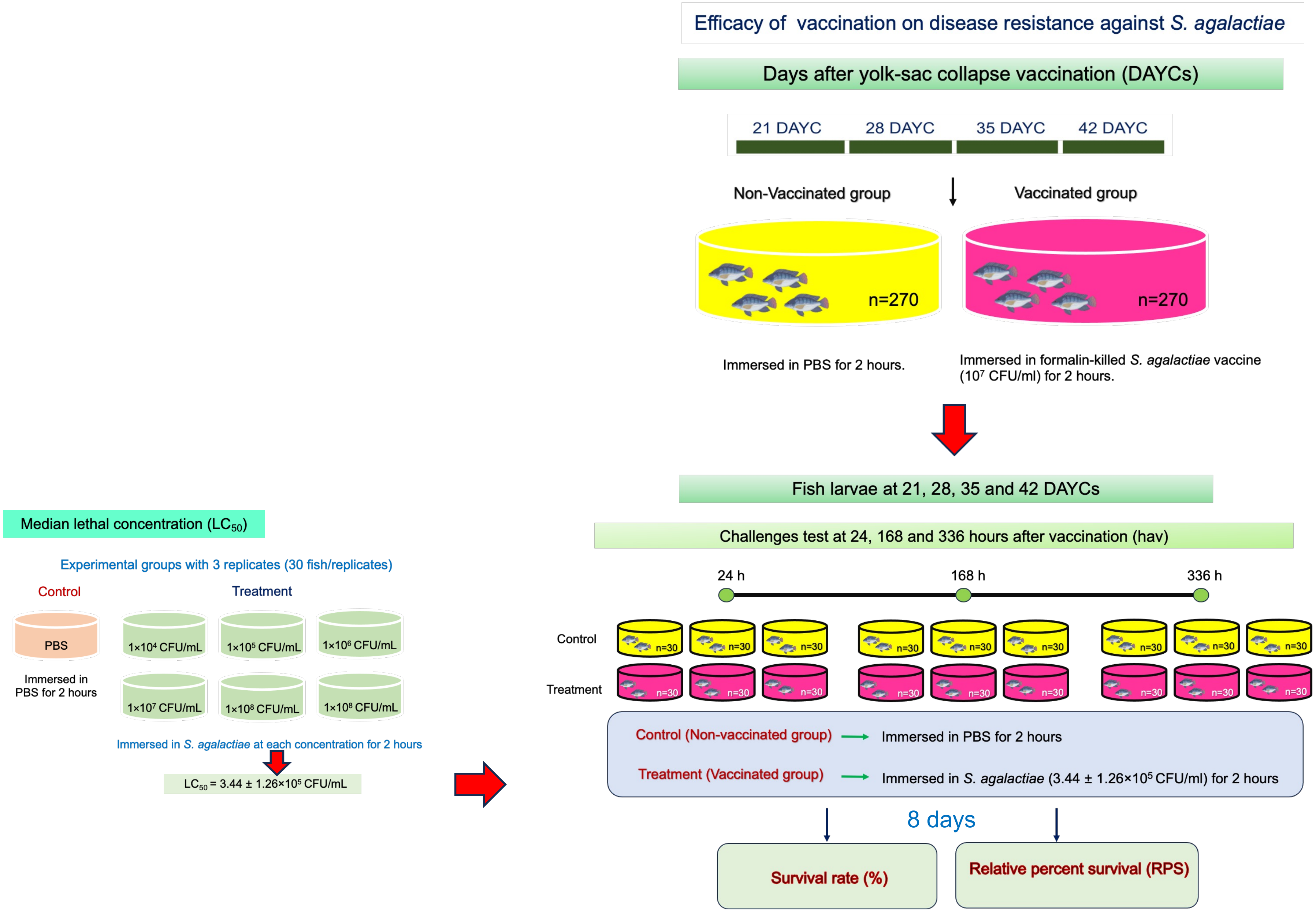

Supplement material Figure S2.
